# Deregulation of phytoene-β-carotene synthase results in derepression of astaxanthin synthesis at high glucose concentration in *Phaffia rhodozyma* astaxanthin-overproducing strain MK19

**DOI:** 10.1186/s12866-019-1507-6

**Published:** 2019-06-15

**Authors:** Lili Miao, Shuang Chi, Mengru Wu, Zhipei Liu, Ying Li

**Affiliations:** 1 State Key Laboratory of Microbial Resources, Institute of Microbiology, Chinese Academy of Sciences, No. 1 West Beichen Road, Chaoyang District, Beijing, 100101 People’s Republic of China; 2 State Key Laboratory of Agrobiotechnology and College of Biological Sciences, China Agricultural University, Beijing, 100193 People’s Republic of China

**Keywords:** *Phaffia rhodozyma*, Glucose metabolism, Astaxanthin, Phytoene-β-carotene synthase, Gene expression

## Abstract

**Background:**

A major obstacle to industrial-scale astaxanthin production by the yeast *Phaffia rhodozyma* is the strong inhibitory effect of high glucose concentration on astaxanthin synthesis. We investigated, for the first time, the mechanism of the regulatory effect of high glucose (> 100 g/L) at the metabolite and transcription levels.

**Results:**

Total carotenoid, β-carotene, and astaxanthin contents were greatly reduced in wild-type JCM9042 at high (110 g/L) glucose; in particular, β-carotene content at 24–72 h was only 14–17% of that at low (40 g/L) glucose. The inhibitory effect of high glucose on astaxanthin synthesis appeared to be due mainly to repression of lycopene-to-β-carotene and β-carotene-to-astaxanthin steps in the pathway. Expression of carotenogenic genes *crt*E, *pbs*, and *ast* was also strongly inhibited by high glucose; such inhibition was mediated by *cre*A, a global negative regulator of carotenogenic genes which is strongly induced by glucose. In contrast, astaxanthin-overproducing, glucose metabolic derepression mutant strain MK19 displayed de-inhibition of astaxanthin synthesis at 110 g/L glucose; this de-inhibition was due mainly to deregulation of *pbs* and *ast* expression, which in turn resulted from low *cre*A expression. Failure of glucose to induce the genes *reg*1 and *hxk*2, which maintain CreA activity, also accounts for the fact that astaxanthin synthesis in MK19 was not repressed at high glucose.

**Conclusion:**

We conclude that astaxanthin synthesis in MK19 at high glucose is enhanced primarily through derepression of carotenogenic genes (particularly *pbs*), and that this process is mediated by CreA, Reg1, and Hxk2 in the glucose signaling pathway.

## Background

Astaxanthin is an orange-red carotenoid pigment that has great commercial value because of its antioxidant and coloration properties. It is utilized increasingly as a protective agent against in vivo oxidative damage [1–5], and as a colorant for aquatic food products such as salmon, lobster, and crab. It can eliminate free radicals with antioxidant activity ~ 500-fold higher than that of vitamin E [6–10]. It has also been reported to inhibit cell toxicity mediated by reactive oxygen species (ROS), to support immune system processes, and to exert anti-aging and anti-cancer effects. For these reasons, there is increasing demand for astaxanthin, particularly from natural sources.

The yeast *Phaffia rhodozyma* (sexual form, *Xanthophyllomyces dendrorhous*; also referred to hereafter as *P. rhodozyma* to avoid confusion) is one of the best natural sources of astaxanthin, because of its capacity for fermentation of glucose via rapid heterotrophic metabolism, and for high cell densities in autofermentors [11–13]. *P. rhodozyma* is a strong fermenter of sugars (e.g., glucose, sucrose, raffinose) -- a desirable property in regard to industrial production. On the other hand, it displays strong Crabtree effect (production of large amounts of fermentation byproducts such as ethanol and acetate, resulting in low biomass), and greatly reduced astaxanthin synthesis at glucose concentrations > 40 g/L [14, 15].

Biosynthesis of carotenoids (tetraterpenoids; a class of terpenoids) is a complicated process, and regulatory mechanisms in *P. rhodozyma* and other producers remain poorly known, although some research progress has been made during the past five years [16–20].

Farnesyl diphosphate (FPP) is a branch-point metabolite of terpenoids and steroids, and CRTE catalyzes formation of geranylgeranyl diphosphate (GGPP) from FPP. This step is generally regarded as the beginning of the carotenoid biosynthesis pathway, because *crt*E (also known as GGPP synthase gene) is part of the carotenogenic gene cluster in all bacteria investigated to date. *crt*E expression is strongly enhanced during fruit ripening, which is accompanied by massive carotenoid formation in *Capsicum* (family Solanaceae) [15]. Formation of phytoene through condensation of two GGPP molecules is catalyzed by phytoene-β-carotene synthase (PBS; encoded by *pbs* gene), a bifunctional enzyme that also displays lycopene cyclase function in *P. rhodozyma* [21, 22]. In four subsequent dehydration steps catalyzed by phytoene dehydrogenase (encoded by *crt*I) [23], double bonds are introduced and lycopene is synthesized. Lycopene is transformed into β-carotene by introduction of ring structures at both ends of the molecule, catalyzed by PBS [22]. β-carotene is hydroxylated and oxidized to astaxanthin by astaxanthin synthase (encoded by *ast*) [24, 27].

Regulation of the astaxanthin biosynthesis pathway in *P. rhodozyma* appeared to occur mainly at the transcript level of carotenogenic genes [25–27], since astaxanthin overproduction was associated with greatly increased expression of these genes. Similar results were obtained for the carotenoid-producing fungi *Neurospora crassa* [28, 29], *Phycomyces blakesleeanus* [30–32], *Mucor circinelloides* [33, 34], and *Fusarium fujikuroi* [35, 36].

Marcoleta et al. observed that the expression of *crt*I, *pbs*, and *ast* in *P. rhodozyma* was inhibited by addition of after adding 20 g/L glucose (20 g/L) to compare with medium, that deplete of glucose [26]. They also showed that the expression *crt*I, *pbs* and *ast* was enhanced by addition of ethanol. They concluded that the astaxanthin repression by glucose was regulated at the transcript level [26]. Pamela et al. reported that transcriptional co-repressor complex Cyc8-Tup1 is involved in regulation of carotenogenesis by glucose [17]. For large-scale astaxanthin production by *P. rhodozyma*, use of high glucose concentration (> 100 g/L) is more practical, in view of the simplified industrial process and less required manpower. We previously established an astaxanthin-overproducing mutant strain of *P. rhodozyma*, termed MK19, by 1-methyl-3-nitro-1-nitrosoguanidine (NTG) and Co60 mutagenesis [37]. MK19 is the most glucose-tolerant *P. rhodozyma* strain reported to date; it maintains its ability to synthesize astaxanthin at glucose concentrations as high as 110 g/L without repressive effect. The regulatory mechanism by glucose in MK19 presumably differs from that in parental (wild-type; WT) strain JCM9042.

In the well-known species *Saccharomyces cerevisiae* (baker’s yeast), regulation of glucose repression is fairly well understood, and the proteins involved have been studied extensively [38]; however, very little is known regarding the glucose signaling mechanism or factors involved in repressor complex structures under high- and low-glucose conditions. No studies along these lines have been performed in *P. rhodozyma*. Glucose repression occurs mainly at the transcriptional level. Accordingly, we investigated deregulation of astaxanthin synthesis by glucose at the transcriptional level and glucose signaling pathway level in JCM9042 and MK19 to clarify the underlying molecular mechanisms.

## Results

### MK19 is a glucose metabolic derepression strain

Both cell growth and astaxanthin synthesis in WT JCM9042 were inhibited by high glucose concentration. Y_X/S_ (biomass production (g) per g glucose) for JCM9042 was reduced from 0.42 at glucose concentration 40 g/L to 0.218 at glucose concentration 110 g/L, and Y_P/S_ (astaxanthin production (mg) per g glucose) was reduced by > 60% from 0.033 (40 g/L) to 0.01(110 g/L) (Table 1). In contrast, no such reduction of Y_P/S_ was observed for MK19.

**Table 1:** Biomass yield and astaxanthin production (per gram glucose) in JCM9042 and MK19

Glucose (g/L)	Y_X/S_ (g/g)^a^			Y_P/S_ (mg/g)^b^		
	40	110	160	40	110	160
JCM9042	0.42 ± 0.003	0.218 ± 0.009	0.136 ± 0.003	0.033 ± 0.003	0.010 ± 0.001	0.003 ± 0.001
MK19	0.405 ± 0.036	0.300 ± 0.004	0.202 ± 0.003	0.250 ± 0.004	0.255 ± 0.001	0.073 ± 0.006

^a^ biomass yield (g) per g glucose. ^b^ astaxanthin production (mg) per g glucose

The low biomass yield in MK19 may have resulted from insufficient oxygen in flasks, leading to incomplete oxidation of glucose by aerobic respiration. Ethanol accumulation was high (> 9 g/L) for 110 g/L glucose, but low for in 40 g/L glucose. Because Crabtree effect can be inhibited by increasing dissolved oxygen level (DO), we reduced medium to 6% liquid volume in flask in order to increase DO. Under this condition, ethanol concentration was much lower than for 10% liquid volume (Table 2).

**Table 2:** Ethanol yield of MK19 as a function of time for various combinations of glucose concentration and liquid volume

	Glucose concentration (g/L)	40	110	110
	Liquid volume	10%	10%	6%
Time (h)	0	0	0	0
	24	0.03	0.244	0.046
	48	0.138	0.807	0.146
	72	0	6.729	0.811
	96	0	9.104	1.317

Biomass yield (cell growth) increased significantly at 6% liquid volume for 110 g/L glucose; Y_X/S_ was 0.382, close to the value for 40 g/L glucose (Table 3). Maximal cell density (OD_600_ > 70), was obtained at 4% liquid volume. At 6% liquid volume, astaxanthin production (Y_P/S_) increased to 0.32 mg/g (Table 3) without change of astaxanthin content. These findings indicate that MK19 is a glucose metabolic derepression strain in regard to both astaxanthin production and cell growth, when DO is sufficient. We used 10% liquid volume for all subsequent experiments because astaxanthin content (μg/g dry weight) did not differ for 6 vs. 10% liquid volume.

**Table 3:** Maximal biomass yield (Y_X/S_) and astaxanthin production (Y_P/S_) in MK19 for various combinations of glucose concentration and liquid volume

Glucose concentration	Liquid volume	Y_X/S_ (g/g)	Y_P/S_ (mg/g)
40 g/L	10%	0.405 ± 0.036	0.25 ± 0.004
40 g/L	6%	0.420 ± 0.016	0.275 ± 0.026
110 g/L	10%	0.300 ± 0.004	0.255 ± 0.001
110 g/L	6%	0.382 ± 0.007	0.320 ± 0.029

### β-Carotene and astaxanthin synthesis in MK19 are not inhibited by high glucose

**Fig. 1:**
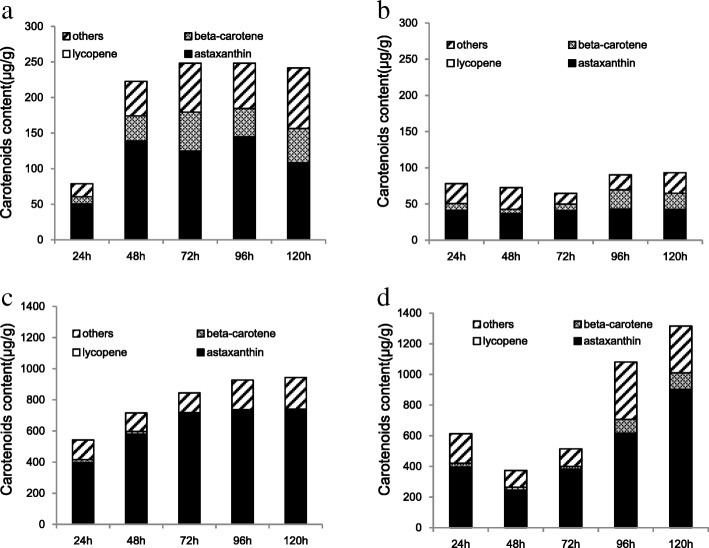
Carotenoid profiles of WT JCM9042 at 40 g/L glucose (**a**), JCM9042 at 110 g/L glucose (**b**), mutant MK19 at 40 g/L glucose (**c**), and MK19 at 110 g/L glucose (**d**)

We examined levels of total carotenoids and pigments other than astaxanthin in cultured JCM9042 and MK19, to investigate regulatory mechanisms of carotenoid biosynthesis by glucose. Total carotenoids and most other pigments were strongly inhibited by high (110 g/L) glucose in JCM9042 throughout the fermentation process; in particular, β-carotene and astaxanthin levels at 72 h were less than 30% of levels at 40 g/L glucose (Fig. 1a,b). β-carotene synthesis increased notably after 72 h, when glucose supply was nearly exhausted (Fig. 1a). Levels of total pigments, β-carotene, and astaxanthin in JCM9042 were much higher for 40 g/L than for 110 g/L glucose, particularly after 48 h when glucose was nearly exhausted (Fig. 1a,b). These findings suggest that high glucose concentration in JCM9042 inhibited synthesis of β-carotene, astaxanthin, and other pigments. Despite notable accumulation of β-carotene, inhibition of astaxanthin synthesis continued even after exhaustion of glucose, indicating differing regulatory mechanisms of the two pigments by glucose.

Transfer of precursors (e.g., GGPP, phytoene) to carotenoids was limited at glucose concentrations > 40 g/L. The reduction of β-carotene (6- to 7-fold) observed for high vs. low glucose from 24 to 72 h was much greater than that of total carotenoids (2- to 3-fold) (Fig. 1). Thus, β-carotene synthesis appears to be the rate-limiting step for astaxanthin synthesis at high glucose concentration.

In stationary phase of growth in MK19, high glucose (110 g/L) caused slight enhancement rather than repression of total carotenoids (1231 μg/g(DW) vs. 937 μg/g(DW) for 40 g/L glucose) and astaxanthin (900 μg/g(DW) vs. 790 μg/g(DW)) (Fig. 1c,d). At 40 g/L glucose, MK19 had astaxanthin level strike greater than that of JCM9042, but lower β-carotene level (Fig. 1), indicating that efficiency of astaxanthin synthase (AST) activity for conversion of β-carotene to astaxanthin was high in MK19 and low in JCM9042. For 110 g/L glucose in comparison to 40 g/L glucose, astaxanthin synthesis was slower during lag and log phases, and excessive astaxanthin was accumulated in stable phase, but final astaxanthin content was similar (Fig. 1c,d). Accumulation of β-carotene was strikingly higher (up to > 50-fold) for 110 g/L vs. 40 g/L glucose. Taken together, these findings suggest that (i) additional AST is required to synthesize more astaxanthin in spite of high *ast* gene expression; (ii) β-carotene synthesis is completely deregulated by high glucose level; (iii) inhibition of astaxanthin synthesis is relieved when glucose is exhausted.

In conclusion, inhibition of astaxanthin synthesis could be more strongly relieved in MK19 than in WT, and such relief resulted from enhanced expression of *ast* gene (Miao et al. 2011). Deregulation of β-carotene synthesis and relief of astaxanthin regulation by glucose lead to excessive β-carotene accumulation in MK19, and further increase of astaxanthin content requires enhancement of *ast* activity.

### Expression of carotenogenic genes *pbs* and *ast* is derepressed in MK19

The pigment profiles described above clearly indicate that repression of astaxanthin biosynthesis pathway by glucose occurs primarily at the lycopene-to-β-carotene reaction step, and to a lesser degree at the β-carotene-to-astaxanthin step in JCM9042. The large reduction of total pigment level under high glucose in JCM9042 suggests that astaxanthin synthesis flux may be blocked at the beginning of carotenoid synthesis; e.g., FPP-to-GGPP or GGPP-to-phytoene step. The deregulation of astaxanthin synthesis by high glucose in MK19 appears to be due mainly to uncontrolled generation of β-carotene from lycopene. To test these possibilities and investigate the molecular mechanisms underlying inhibition of astaxanthin synthesis in *P. rhodozyma*, we examined expression of carotenogenic genes under high vs. low glucose concentration in JCM9042 and MK19.

**Fig. 2:**
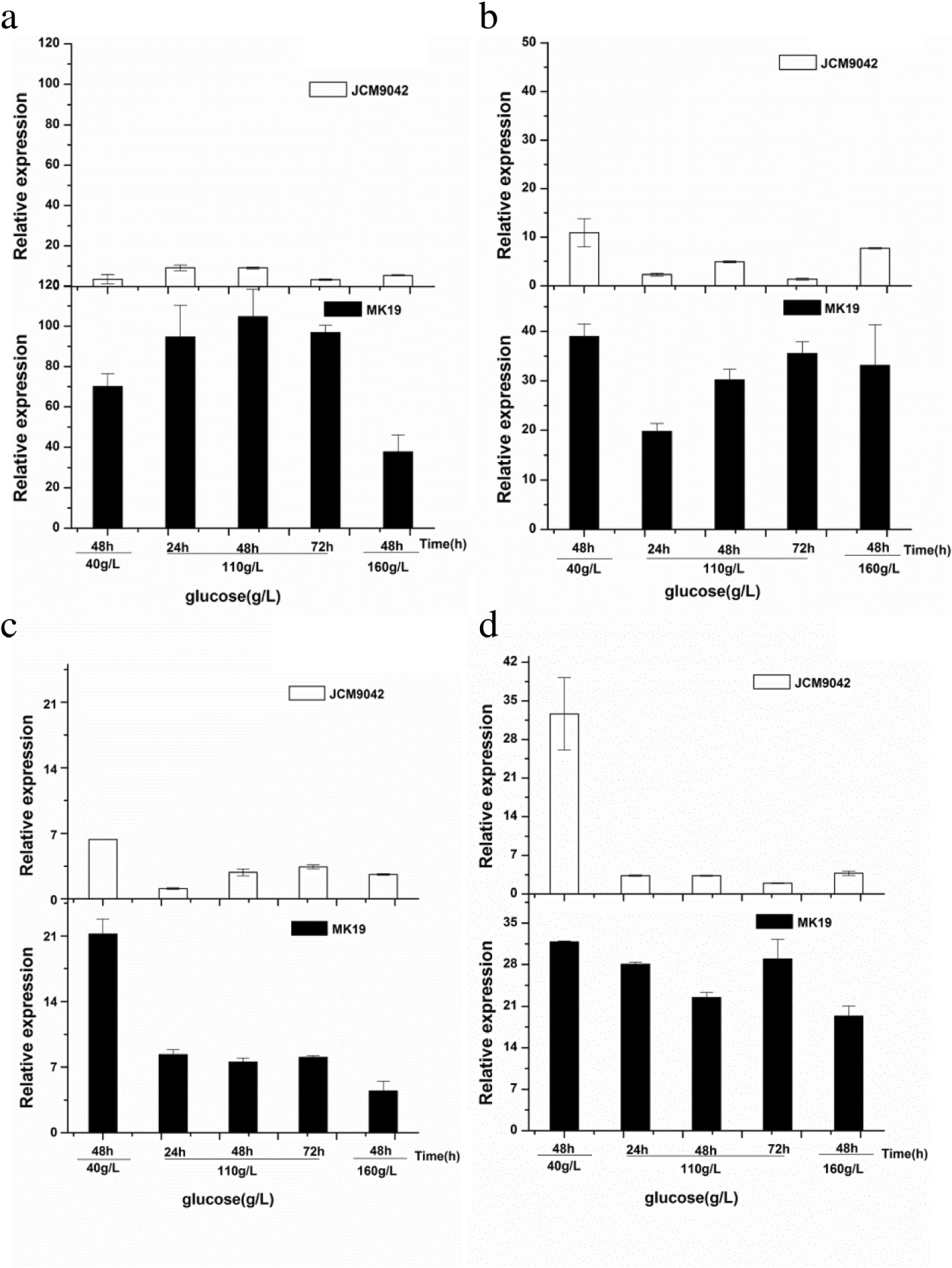
Relative expression of *crt*I (**a**), *ast* (**b**), *crt*E (**c**), and *pbs* (**d**) genes at glucose concentrations of 40, 110, and 160 g/L

Expression of carotenogenic genes was fairly stable throughout cultivation at 110 g/L glucose except for *ast*, which showed increased mRNA until 48 h (Fig. 2). Therefore, a positive effect of ethanol on transcription of carotenogenic genes can be ruled out (Table 2) [26], and we used only log phase (48 h) samples from 40 and 160 g/L glucose conditions for comparisons of gene expression.

JCM9042 showed much lower mRNA level for structural gene *ast* at 110 g/L than 40 g/L glucose (Fig. 2b), consistently with the findings of Marcoleta et al. [26], and mRNA level at 72 h under 110 g/L glucose was ~ 30% of that at 48 h. This finding may account in part for the observation that astaxanthin level did not increase even after exhaustion of glucose (72 h) (Fig. 1), and the low proportion (40%) of astaxanthin among total carotenoids in JCM9042 at 110 g/L glucose. In MK19, glucose concentration did not notably affect *ast* expression except at 24 h. In both JCM9042 and MK19, *crt*I transcription was higher at 110 than at 40 g/L glucose, but was reduced at 160 g/L glucose (Fig. 2a). In contrast, Marcoleta et al. [26] found that *crt*I was downregulated even at low glucose concentration. In JCM9042 at 110 g/L, enhancement of *crt*I mRNA was correlated with accumulation of lycopene, whose production is catalyzed by the enzyme phytoene desaturase (CrtI).

In JCM9042, mRNA transcription of *crt*E and *pbs* declined rapidly as glucose concentration increased (> 40 g/L), suggesting that repression of astaxanthin by glucose occurs mainly during synthesis of GGPP, or of phytoene and lycopene. This concept is supported by the pigment profile of JCM9042, the reduction of total pigment level during cultivation, and the low proportion of β-carotene from 24 to 72 h at 110 g/L glucose, when glucose is still abundant. In MK19, *pbs* was completely deregulated by glucose (Fig. 2d). *crt*E expression was inhibited by high glucose in MK19, even though *crt*E mRNA level was > 2-fold higher than that of JCM9042 (Fig. 2c). This finding may account for the slow synthesis of total carotenoids in MK19 at 110 g/L glucose prior to log phase. The enhanced *crt*E mRNA level in MK19 relieved the inhibition of precursor-to-pigment synthesis, and in combination with deregulation of *pbs* gene resulted in final astaxanthin level similar to that at low glucose. It is also suggested that further increase of *crt*E mRNA may further enhance astaxanthin content. Uncontrolled astaxanthin synthesis at high glucose was presumably due mainly to deregulation of *pbs*.

### *cre*A expression is not induced by high glucose in MK19

**Fig. 3:**
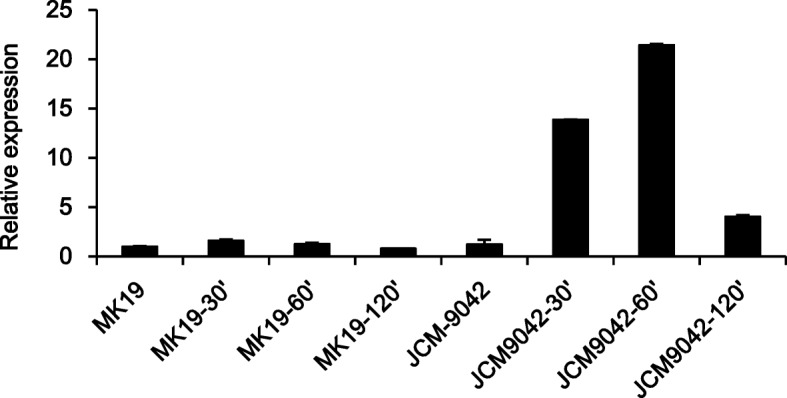
Relative expression of *cre*A in MK19 and JCM9042 at glucose induction times 0, 30, 60, and 120 min as indicated in the X-axis captions

Carotenogenic genes are regulated by glucose through protein CreA, a global negative regulator. We examined CreA expression to evaluate the possibility that astaxanthin synthesis is controlled by glucose through downregulation of *pbs* expression. The role of *cre*A has been intensively was studied in *S. cerevisiae* [38]. Moreno et al. [38] found that high glucose induced *cre*A gene expression in *S. cerevisiae*. Cifuentes et al. [16] showed that carotenogenic synthesis gene promoters contained a CreA binding position, and used electrophoretic mobility shift assays (EMSAs) to improve the interactions of these genes with CreA. In both JCM9042 and MK19, *cre*A mRNA was at a similar low level when glucose was exhausted after 48 h culture. In JCM9042 at 110 g/L glucose, *cre*A expression was strongly induced at 60 min, whereas *cre*A mRNA in MK19 remained at a similar low level regardless of glucose induction time (Fig. 3).

**Fig. 4:**
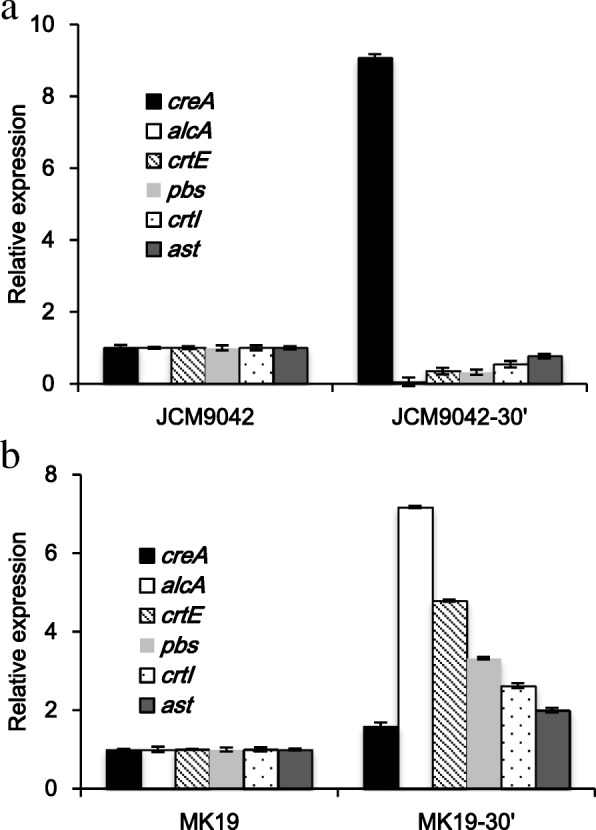
Relative expression of *cre*A, *alc*A, and carotenoid synthesis genes in JCM9042 (**a**) and MK19 (**b**) at glucose induction times 0 and 30 min. Expression at 48 h was defined as 1. *alc*A, encoding the alcohol dehydrogenase I and repressed by *cre*A, was used as control

Expression of *alc*A, *crt*E, and *pbs* at glucose induction time 30 min was suppressed in JCM9042 (Fig. 4a), but enhanced in MK19 (Fig. 4b). Thus, deregulation by glucose of carotene synthesis genes in MK19 appears to result from failure induction of the global negative regulator CreA.

**Fig. 5:**
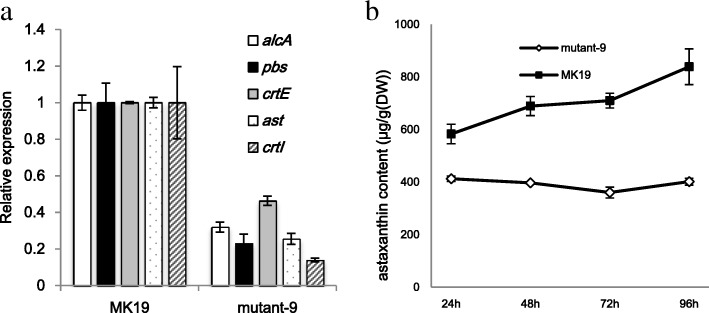
(**a**) Relative expression of *alc*A and carotenoid synthesis genes in MK19 and MK19-CreA9 (mutant 9) at 24 h at 40 g/L glucose. Expression in MK19 was defined as 1. (**b**) Astaxanthin content of MK19 and mutant 9 as a function of fermentation time

To test the concept that derepression of astaxanthin synthesis genes such as *pbs* in MK19 resulted mainly from low *cre*A mRNA, we expressed extra copies of this gene. The transformed clone MK19-CreA9 (termed “mutant 9”) showed a distinct pink color, particularly at early culture stages (before 72 h), when glucose was abundant, even though initial glucose concentration was low (40 g/L in seed medium, the mutants cannot grow in synthesized medium.). Total carotenoid content of mutant 9 was only ~ 50% that of MK19 (Fig. 5b). In comparison with MK19, mutant 9 showed slower cell growth prior to 72 h, and much lower expression of *pbs*, *ast*, *crt*E, *crt*I, and *alc*A genes (Fig. 5a).

These findings indicate that derepression by glucose of astaxanthin synthesis in MK19 was due to the fact that *cre*A was not induced by glucose, and consequently did not inhibit expression of its target genes (*pbs*, *ast*, etc.).

### Expression of *reg*1 and *hxk*2 in MK19 glucose signaling pathway genes was not induced by glucose

**Fig. 6:**
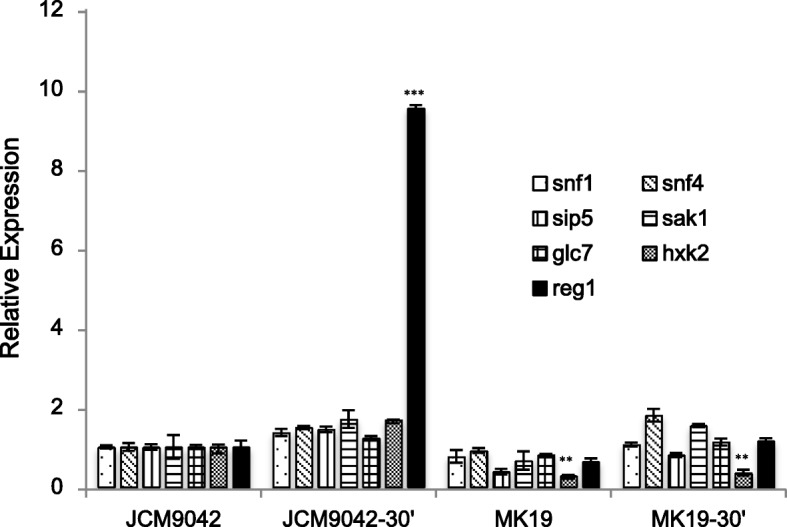
Relative expression of glucose signaling pathway genes in MK19 and JCM9042 at glucose induction times 0 and 30 min. Expression in wild type JCM9042 at 48 h was defined as 1

We measured expression of glucose signaling pathway genes (*snf*1, *snf*4, *sip*5, *sak*1, *glc*7, *hxk*2, *reg*1) to elucidate the molecular mechanisms underlying regulation of astaxanthin synthesis by glucose in *P. rhodozyma*. The protein CreA is regulated by Snf1 kinases and Glc phosphatase. At high glucose level, Snf1 is inactive and cannot phosphorylate CreA. When glucose level falls, Snf1 becomes active and phosphorylates CreA, which cannot then bind to target genes such as *alc*A and carotenogenic genes. High glucose also inactivates Snf1 by stimulating activity of Glc7-Reg1 phosphatase. Presence of Hxk2 in the repressor complex is necessary for inhibition of Snf1 kinase-induced CreA phosphorylation [38]. Addition of glucose promotes Hxk2/ Reg1 interaction and inactivation of Snf1. *hxk*2 mRNA level in MK19 was much lower than in JCM9042, and *hxk*2 expression was not induced by glucose (Fig. 6). This is one reason why MK19 was unable to maintain CreA in active state and showed downregulation of carotenogenic genes at high glucose level. In JCM9042 at 110 g/L glucose, *reg*1 mRNA level was much higher (~ 10-fold) at glucose induction time 30 min than at 0 min. In contrast, *reg*1 mRNA level in MK19 was only slightly higher at 30 min than at 0 min (Fig. 6). *sak*1 gene expression, which promotes Snf1 kinase activity at low glucose level, was induced by glucose in MK19. These findings indicate that failure of glucose to induce *hxk*2 and *reg*1 explains why astaxanthin synthesis in MK19 was not inhibited at high glucose level.

## Discussion

*Phaffia rhodozyma* is a preferred industrial strain for production of natural astaxanthin because of several desirable properties, e.g., synthesis of free astaxanthin as principal carotenoid in the absence of light, fermentation of many types of saccharides, and high cell density in autofermentors. Typically, cell growth and astaxanthin synthesis of *P. rhodozyma*, which displays positive Crabtree effect, are inhibited by high glucose concentration. We previously established a moderate-temperature, high-glucose-tolerant, astaxanthin-overproducing *P. rhodozyma* strain, termed MK19 [37]. Astaxanthin synthesis by MK19 is not repressed by high glucose level (up to 110 g/L). In contrast, cell growth and carotenoid production of WT strain JCM9042 can only tolerate glucose level up to 40 g/L. MK19 is a glucose metabolic derepression strain in regard to both astaxanthin production and cell growth, when DO is sufficient. *hxk*2 mRNA level in MK19 is low (see Results), consistent with the previous finding of reduced Crabtree effect in an *hxk*2 mutant of *S. cerevisiae*, resulting in nearly complete respiratory metabolism at high glucose level [38].

### Repression of astaxanthin synthesis by high (110 g/L) glucose in *P. rhodozyma* depends mainly on bifunctional enzyme phytoene-β-carotene synthase (PBS)

The reduced levels of β-carotene, astaxanthin, and total pigments observed at 110 g/L glucose in JCM9042 indicate that repression occurs at the lycopene-to-β-carotene step, β-carotene-to-astaxanthin step, or even beginning of the carotenoid biosynthesis pathway (GGPP formation). Levels of β-carotene and total carotenoids were much lower (11–14%) at 110 than at 40 g/L glucose, suggesting that β-carotene synthesis is the rate-limiting step for astaxanthin synthesis at high glucose level. In contrast, synthesis of total carotenoids and astaxanthin was not controlled by high glucose during MK19 stable phase. At 40 g/L glucose, astaxanthin content was 10-fold higher whereas β-carotene was much lower in MK19 than in JCM9042, indicating that β-carotene-to-astaxanthin conversion efficiency was high in MK19 but very low in JCM9042. In MK19, β-carotene synthesis was not inhibited by 110 g/L glucose; final astaxanthin content was similar at 40 vs. 110 g/L glucose, and β-carotene accumulation at 120 h was ~ 50-fold higher at 110 g/L glucose. Additional AST activity was evidently necessary to further increase astaxanthin production, even though *ast* is highly expressed in MK19 [27]. Metabolic engineering (overexpression) of *ast* is a useful approach for further enhancing astaxanthin production in MK19 [39]. These conclusions are consistent with observed expression of structural genes (*crt*E, *pbs*, *ast*, *crt*I) in JCM9042 and MK19 at various glucose concentrations (Fig. 2).

Transcription of *crt*E and *pbs* in JCM9042 was greatly inhibited at glucose concentrations > 40 g/L; in particular, *pbs* was reduced to 10% at 110 g/L glucose. These findings suggest that repression of astaxanthin by glucose metabolites in *P. rhodozyma* also occurs at the GGPP, phytoene, and lycopene synthesis steps. This concept is supported by the consistency between expression profiles and pigment profiles in JCM9042.

Although *crt*E mRNA level was 2-fold higher in MK19 than in JCM9042, this gene was still inhibited by high glucose, resulting in slower accumulation of pigments prior to log phase. Final total pigment content was higher at 110 than at 40 g/L glucose, even though *crt*E mRNA level was lower at 40 g/L glucose. GGPP is a point metabolite that connects carotenoids to other isoprenoids. High carotenoid synthesis, stimulated by high-glucose-induced deregulation of *pbs* and *ast*, presumably redistributes GGPP from other isoprenoids biosynthesis pathways to carotenoid flux pathway in MK19, leading eventually to similar final astaxanthin content at low and high glucose levels. We conclude that uncontrolled astaxanthin synthesis at high glucose in MK19 results mainly from deregulation of *pbs* and (to a lesser degree) *ast* gene, leading to high transfer efficiency from GGPP to phytoene, lycopene to β-carotene, and β-carotene to astaxanthin.

### Repression of *pbs* by glucose is mediated by *cre*A, *reg*1, and *hxk*2 in the glucose signaling pathway

CreA, the transcription factor primarily responsible for repression of genes necessary for utilization of alternative fermentable carbon sources, binds to DNA and inhibits transcription of target genes. Snf1 kinase plays a key role in regulation of the glucose repression signaling pathway. The relationships among glucose level, Snf1 kinase activity, CreA phosphorylation, Glc7-Reg1 phosphatase, *alc*A, carotenogenic genes, and Hxk2/ Reg1 interaction are described in Results/ “Expression of *reg1* and *hxk2* in MK19 glucose signaling pathway genes ...”. Upregulation of genes with CreA binding sites has been reported in *hxk*2-deletion strains [38].

The role of *cre*A has been intensively studied in *S. cerevisiae* [38]. In both JCM9042 and MK19, *cre*A mRNA was at similar low levels when glucose was exhausted after 48 h culture, whereas its level was strikingly different in the two strains at 110 g/L glucose with 60 min induction time (Fig. 3). At 30 min glucose induction time, JCM9042 showed repression of *alc*A, *crt*E, and *pbs*, together with strong induction of *cre*A, whereas MK19 did not show *cre*A induction or repression of its target genes. Thus, deregulation by glucose of carotenoid synthesis genes in MK19 is evidently due to failure of global negative regulator CreA. This concept is supported by the observed downregulation of *alc*A, *crt*E, *pbs,* and *ast* in *cre*A-overexpressing strain MK19-CreA9 (mutant 9).

To clarify the molecular mechanisms underlying regulation of astaxanthin synthesis by glucose in JCM9042 and MK19, we measured expression of glucose signaling pathway genes as described under Results/ “Expression of *reg1* and *hxk2* in MK19 glucose signaling pathway genes”. *hxk*2 mRNA level was much lower in MK19 than in JCM9042, and was not induce by glucose (Fig. 6). *reg*1 mRNA level in JCM9042 at 110 g/L glucose was ~ 10-fold higher at induction time 30 min than at 0 min, but in MK19 was only slightly higher at 30 min than at 0 min. Low *hxk*2 and *reg*1 mRNA levels accounted for the failure of MK19 to maintain CreA active state, and for downregulation of carotenogenic genes at high glucose concentration. In contrast, expression of *sak*1, which promotes Snf1 kinase activity when glucose is exhausted, was enhanced in MK19 at 110 g/L glucose. Thus, failure of glucose to induce *hxk*2 and *reg*1 accounts for the fact that astaxanthin synthesis in MK19 was not repressed at high glucose.

**Fig. 7:**
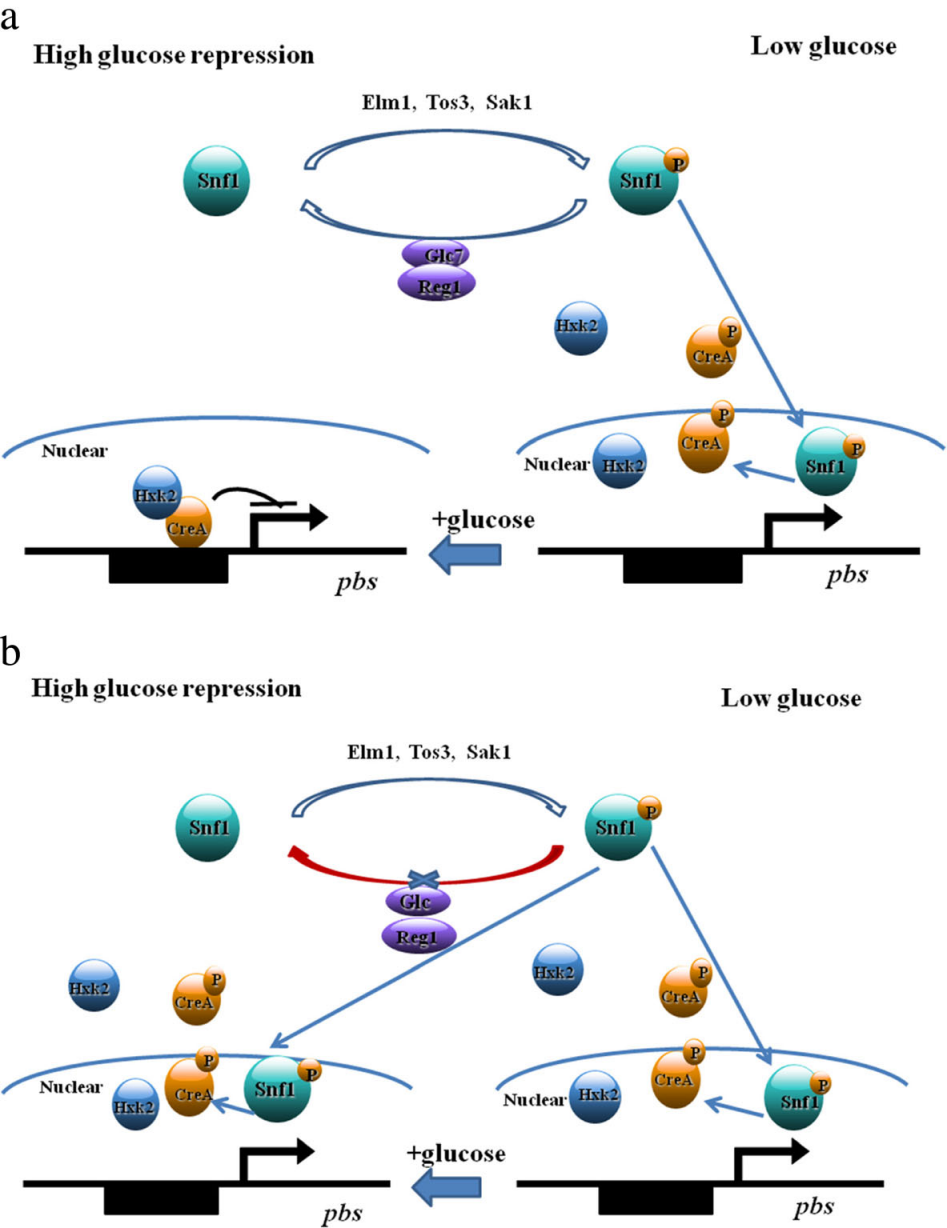
Regulatory pattern of glucose signaling pathway on *pbs* in JCM9042 (**a**) and MK19 (**b**)

This study reports the involvement of *reg*1 and *hxk*2 in regulation of carotenogenesis in yeast. A proposed regulatory pattern of glucose signaling pathway on *pbs* in JCM9042 and MK19 is shown schematically in Fig. 7.

The bifunctional enzyme PBS catalyzes generation of phytoene and β-carotene. Secondary metabolites (e.g., carotenoids) often display high synthesis/ accumulation in adverse (nutritionally deficient) environments. A simple method whereby microorganisms can reduce flux of secondary metabolites is to control multifunctional enzymes responsible for multiple pathway steps under good-nutrition conditions. Glucose at high concentration controls astaxanthin synthesis in *P. rhodozyma* primarily by inducing expression of glucose signaling pathway genes (*reg*1, *hxk*2) that maintain activity of *cre*A and repress its target gene *pbs*. Thus, engineering of *pbs*, *cre*A, *reg*1, and/or *hxk*2 is potentially an efficient approach for increasing astaxanthin production on an industrial scale.

In summary, repression by glucose of astaxanthin in *P. rhodozyma* occurred mainly in the pathway steps for generation of GGPP, phytoene, lycopene, and astaxanthin, and was based on control of *pbs, ast,* and *crt*E transcription. Derepression of astaxanthin synthesis at high glucose level in astaxanthin-overproducing strain MK19 was due mainly to deregulation of *pbs, ast*, and glucose signaling genes *reg*1, *hxk*2, and *cre*A, leading to enhanced efficiency of GGPP-to-phytoene and lycopene-to-astaxanthin transfers.

## Methods

### Strains and culture conditions

WT *P. rhodozyma* strain JCM9042 was obtained from the Institute of Physical and Chemical Research (RIKEN), Wako, Japan. The highly pigmented mutant strain MK19 was established by NTG and Co60 mutagenesis in our laboratory (Miao et al. 2010). Both strains were maintained on potato dextrose agar slants at 4 °C.

Seed medium and fermentation medium were prepared as described previously [27]. Experiments were performed in shaking flask culture, with 250-mL flasks containing liquid volume 25 mL. YPD medium:Glucose 20 g/L, yeast extracts 10 g/L, peptone 20 g/L.

WT and mutant cells were transferred from 4 °C slants to fresh slants, and kept for 72 h at 21–24 °C. Loopfuls of lawn were inoculated to seed medium, and incubated for 72 h at 21–24 °C. Starter culture was produced by inoculation of 5% pre-incubation broth for an additional 36 h. Overall production period of fermentation culture was 4–6 days on a rotary shaker (210 rpm), 21 °C, with samples taken at 12-h or 24-h intervals. Experiments were performed in triplicate or quadruplicate.

Cell concentration was estimated as OD_600_. Dry weight of cells was determined by centrifuging 35 mL broth at 12,000 rpm, rinsing with distilled water, and drying at 85 °C to constant weight (~ 15 h).

### Measurement of astaxanthin

1 mL broth was centrifuged at 12,000 rpm for 1 min, and washed with distilled water. Pellets were mixed with 200 μL dimethyl sulfoxide preheated to 70 °C, stirred, and the mixture was maintained at 70 °C in a waterbath for 20 min. Broken cells were extracted with methanol/ dichloromethane (3:1), agitated, and centrifuged at 2000 rpm. Supernatant was transferred to another tube. This process was repeated until pellets showed no red color. Astaxanthin and free ergosterol were analyzed quantitatively by HPLC on a C18 column (250 × 4.6 mm; 5 μm; Chuangxintongheng Science & Technology Co., Beijing): temperature 40 °C, flow rate 1.0 mL/min, wavelength 476 nm for astaxanthin or 280 nm for ergosterol. Mobile phase consisted of methanol 97%, water 3%. Astaxanthin and ergosterol were identified based on retention time relative to standard astaxanthin (Sigma).

### Total RNA purification, reverse transcription, and real-time PCR (RT-PCR)

Total RNA purification and reverse transcription were performed as described previously [27, 37]. RT-PCR analyses were performed using an ABI 7900HT system (Applied Biosystems; Norwalk, CT, USA) with RNA samples as template. Dissociation curves were constructed to confirm amplification. Target genes were from NCBI (ncbi.nlm.nih.gov); database accession numbers and corresponding primer sets in RT-PCR were as described previously [27]. *actin* was used as control gene. Relative gene expression was calculated by 2^-***△△****CT*^ (cycle threshold) method using Sequence Detection System software program v1.2.2 (Applied Biosystems). Each RT-PCR analysis was run in triplicate or quadruplicate to test consistency.

### Construction of MK19 *cre*A-overexpressing strain

Plasmid for expression of *cre*A was constructed in pGBKT7 and transformed to MK19 according to Chi et al. [39]. 40 μg/ml G418 in YPD medium was used to screen positive transformants. Plasmids of each transfromant were extracted using a yeast plasmid extraction kit (Tiangen Biotech; Beijing, China) and identified by PCR using primers *cre*AP (5′-AAGACCTTGACATGATTTTGAA-3′) and *cre*AC (5′-GGACACTTGTACGGCCTG-3′). The characterization and stable of mutants were also according to Chi et al. [39].

## Data Availability

All data and materials are available upon request.
